# AI alignment is all your need for future drug discovery

**DOI:** 10.3389/frai.2025.1668794

**Published:** 2025-11-03

**Authors:** Chunyan Li

**Affiliations:** School of Informatics, Yunnan Normal University, Kunming, China

**Keywords:** AI alignment, drug discovery, human values, generative AI, robustness, interpretability

## Abstract

In recent years, the integration of artificial intelligence (AI) with drug discovery has become a promising frontier in biomedical research. However, as artificial intelligence systems become increasingly complex, ensuring their alignment with human values and goals becomes essential. Specifically, combining artificial intelligence systems with human values is crucial for reducing potential risks in the field of drug discovery and maximizing social benefits. This article explores the concepts and challenges related to alignment with artificial intelligence in the context of drug discovery, emphasizing on human-centered approaches to AI development and deployment. We further investigated popular technology frameworks designed for human-centered AI alignment, aimed at improving the robustness and interpretability of AI models. We provide some insights into the challenges of human-centered AI alignment, which represents a significant advancement in addressing robustness and interpretability, thus taking a step forward in the field of AI alignment research. Finally, we discuss strategies for systematically integrating human values into AI-driven drug discovery systems. This article aims to emphasize the importance of AI alignment as a foundational principle in the field of drug discovery and advocate the perspective that “AI alignment is all your need for future drug discovery”.

## 1 Introduction

The annals of human civilization are replete with narratives of endeavors to combat afflictions and calamities. Essential to this effort are pharmaceuticals, which constitute the principal ways for mitigating diseases, having been progressively refined through human empirical inquiry and application. Nonetheless, conventional drug development methods still face significant challenges, including high costs, lengthy timelines, and meager success rates. In fact, the diffcult road from drug conceptualization to market outcomes typically spans ten years, with expenditures approaching 2.6 billion dollars. In addition, the success rate of candidate drugs transitioning from trial phases to market availability scarcely exceeds 10% (Avorn, 2015; DiMasi et al., 2016). Hence, artificial intelligence has significantly shortened the drug development trajectory, provided novel avenues for the advancement of drug discovery (Wu et al., 2018; Xia et al., 2023; Li et al., 2022a, 2021b, 2023).

However, with the continuous improvement of artificial intelligence (AI) system capabilities, related risks are also increasing (Ji et al., 2024). Emerging trends, such as the proliferation of intelligent agents based on large language models and the development of generative AI technology, have the potential to achieve a form of universal artificial intelligence where systems may attain or even surpass human-level intelligence in specific domains. Although these advancements foreshadow potential benefits such as automation, increased efficiency, and accelerated technological progress, they also bring significant risks, including security vulnerabilities, biases, and societal inequalities. Moreover, there are concerns about the potential impact of large-scale deployment of superhuman artificial intelligence systems. Notably, contemporary large language models exhibit significant biases in terms of gender, sexual identity, and immigration status, which exacerbates pre-existing social inequities. Furthermore, the negative behaviors observed in these models, such as the propagation of inaccurate responses, flattery, and deceit, tend to escalate with the amplification of model size, which raises ethical considerations regarding the deployment of advanced artificial intelligence systems. Concurrently, the development of generative language models has sparked discussions around the control and governance mechanisms, which is required for effective management such systems (Arora, 2024).

In the domain of drug discovery (Urbina et al., 2022), if the output provided by large language models is proven to be erroneous or fallacious, it has the potential to misguide researchers and trigger erroneous determinations throughout the entire drug discovery process, which, in turn, may lead to resource waste, deviation from the intended research trajectory, and the inadvertent introduction of unsafe or inefficacious drugs into the market. Large language models may be susceptible to manipulation or exploitation as a channel for the promotion of specific drugs or therapeutic modalities, regardless of their empirical foundation or clinical efficacy. Such instances of flattery and deceit have the propensity to compromise patient welfare, which will lead to the adoption of superfluous or ineffectual treatment methods. The sensitivity of large language models to data-driven biases can exacerbate bias in research outcomes and drug recommendations, which could exacerbate existing healthcare inequalities. In scenarios where large language models provide illegal or morally questionable recommendations, such as the promotion of prohibited substances or improper drug utilization, which will arise legal culpability and moral quandaries, thereby questioning the integrity and credibility of the healthcare industry. Hence, the harmful behaviors exhibited by large language models indicate deleterious impacts on research, clinical practice, and patient welfare (Urbina et al., 2022; Vijayan et al., 2022).

In the context of AI-driven drug discovery, human values refer to fundamental principles such as fairness, transparency, accountability, and respect for human well-being, which are key guiding principles. Embedding these values into artificial intelligence systems is not only a moral requirement, which is also a practical necessary condition to ensure that drug discovery results are trustworthy, beneficial to society, and meet the needs of patients. For example, fairness is crucial in preventing bias in data analysis and candidate prioritization. Transparency improves the interpretability of predictive models. This multidimensional perspective emphasizes that combining artificial intelligence systems with human values can greatly impact the credibility and acceptance of society. Han et al. (2022) further emphasized the need for a deep understanding of the consistency between artificial intelligence and human values, providing important insights on how to achieve this consistency in the biomedical field. Therefore, the integration of human-centered artificial intelligence (HCAI) into drug discovery offers several potential benefits (Mbatha et al., 2023). By aligning AI systems with human values, there is a greater focus on ensuring that drug candidates identified by AI are safe and effective for human use, which could reduce adverse effects and improve the success rate of clinical trials (Wang et al., 2023). Human-centered AI can help ensure that drug discovery efforts are inclusive and meet the needs of different populations (Zheng et al., 2023). Integrating human values into AI systems can ensure that ethical considerations are central to the drug discovery process. By prioritizing human-centered approaches, AI systems can mitigate potential biases in the data or algorithms used in drug discovery, which leads to more fair and unbiased decision-making processes, ultimately improving the fairness and reliability of drug discovery efforts. Human-centered AI alignment can simplify the drug discovery process by prioritizing drug candidate drugs that are most likely to meet the needs and preferences of patients and clinical doctors, which can lead to faster development timelines and more efficient resources allocation in drug discovery research.

The endeavor to achieve AI alignment depict the key basic trajectory toward the attainment of human-centered AI. AI alignment (Ji et al., 2024) is predicated on the imperative to engender AI systems that align their behavior with human intentions and values. This pursuit includes four overall objectives, namely Robustness, Interpretability, Controllability and Ethicality (RICE) (Ji et al., 2024). AI alignment has the potential to fundamentally change the drug discovery process by improving efficiency, safety, ethical considerations, and fairness. By aligning AI systems with human intentions and values, researchers can harness the transformative power of AI to address unmet medical needs and improve patient outcomes in a responsible and equitable manner. Ji et al. believe that as the capabilities of artificial intelligence systems continue to increase, the risk of alignment failure is also increasing. Mitigating the extinction risk brought by artificial intelligence should become an important global priority. They first proposed four key objectives for AI alignment, namely RICE, and divided AI alignment into two key components: forward alignment and backward alignment (Ji et al., 2024). The difference in this article's approach is that we provide a review of the alignment problem of artificial intelligence in the field of drug discovery, with a focus on robust and interpretable technical architectures.

To push the boundaries of drug discovery into human-centered AI alignment as shaping a positive future of drug discovery, we argue that aligning AI systems with human values is essential to mitigate potential risks and maximize societal benefits in the domain of drug discovery. In this paper, initially, we scrutinized the challenges inherent in AI alignment. Subsequently, we survey popular technical frameworks designed for human-centered AI alignment and give some insights to aim at enhancing the robustness and interpretability of artificial intelligence models, that are the two most critical objectives of AI alignment. Finally, we propose relevant strategies to incorporate human values into AI systems for drug discovery. In summary, the key contributions of our work are as follows:

- To the best of our knowledge, this work represents the first comprehensive survey of technical frameworks specifically designed for human-centered AI alignment, with a focus on AI technologies in the field of drug discovery.
- We give some insights into the challenge of AI alignment, aimed at enhancing the robustness and interpretability of artificial intelligence models based on molecule-related downstream tasks, which describes our preliminary findings concerning AI alignment.
- We propose relevant strategies to incorporate human values into the design and implementation of AI systems for drug discovery.

## 2 Challenges in AI alignment

Foundation models, such as GPT-3 (Brown et al., 2020) and BERT (Devlin et al., 2019), can advance the development of science by augmenting research methods with foundation models and generative AI. If explicit guidelines are formulated for the utilization of foundation models and generative AI, the potential benefits they offer to science and scholarly inquiry outweigh the associated risks (Rossi et al., 2024). The endeavor to construct artificial intelligence systems that harmonize with human values and intentions poses a significant challenge. Presently, there lacks a universally accepted standard for gauging alignment. Leike et al. (2018) have described the intelligent agent alignment problem and expanded its scope to encompass super artificial intelligence systems (OpenAI, 2023b). Meanwhile, the RICE principle proposed by Ji et al. (2024) centers on discerning and accommodating human intentions, which points the four cardinal objectives of AI alignment: Robustness, Interpretability, Controllability and Ethicality. These four basic objectives form a unified whole, referred to as the RICE principles, which present distinct challenges in the context of AI alignment.

- **Robustness:** The robustness of AI systems represents their capacity to maintain stability and dependability amid diverse uncertainties, disruptions, or adversarial attacks (Dietterich, 2017). In essence, a robust AI system excels at maintaining proficient performance across varied environments and situations, while exhibiting robust adaptability to alterations or perturbations in input data. A robust AI system exhibits minimal performance degradation or susceptibility to failure when confronted with variations in external conditions. Such robustness constitutes a pivotal attribute requisite for ensuring the dependable operation of AI systems in real-world settings and for effectively addressing various challenges. To enhance the robustness of AI systems, researchers typically employ a variety of methods, including but not limited to augmenting the diversity of training data, implementing data augmentation techniques, improving algorithms and model architectures for enhanced resilience, and designing tailored testing and evaluation protocols. By iteratively enhancing the robustness of the system, it becomes better equipped to navigate complex real-world scenarios, thereby enhancing the efficacy and reliability of AI systems in different application domains. Aligned systems always consistently maintain robustness throughout its entire lifecycle (Russell, 2019).
- **Interpretability:** The interpretability of AI systems involves their ability to provide clear and transparent explanations or reasoning, thereby facilitating user comprehension of the system's operational framework, decision-making mechanism, and underlying principles for recommendations. A well interpretable AI system can explain its behavior and provide reasonable reasons for its decisions, rather than merely providing results. In many application scenarios, people not only need accurate predictions or decisions from AI systems but also need to understand the mechanisms that support these results and the basic rationale that drive such decisions. Interpretability is crucial in cultivating user's confidence in the system, augmenting its credibility, and aiding in the identification of potential biases or discrepancies therein. Furthermore, the interpretability of AI systems helps clarify the decision-making logic for users and facilitates system developers to identify and correct potential defects, thereby enhancing the robustness and reliability of the system. Therefore, interpretability has emerged as a pivotal research approach within the realm of artificial intelligence, attracting widespread attention and applicability in different practical areas (Lipton, 2016; Guidotti et al., 2018; Doshi-Velez and Kim, 2017).
- **Controllability:** The controllability of AI systems refers to the ability of humans to effectively manage and control the behavior and decision-making of the system, ensuring that the system's actions and decision-making processes are always supervised and constrained by humans (Soares et al., 2015; Hadfield-Menell et al., 2017). Controllability focuses more on real-time human intervention and adjustment of the system to ensure that it meets human expectations and needs during operation, while avoiding adverse or unexpected consequences. With the increasing development of AI technology, more and more research has expressed concerns about the controllability of these powerful systems (Critch and Krueger, 2020). So, the controllability of AI systems encompasses the capacity to fulfill human expectations and objectives while mitigating potential risks and uncertainties (Bowman et al., 2022).
- **Ethicality:** The ethical behavior of AI systems is related to their capacity to comply with ethical norms and values during the design, development, deployment, and utilization phases, which includes ensuring that the behavior and decisions of the system comply with ethical norms, upholding individual rights and dignity, and exhibiting accountability toward societal and public interests (Floridi and Sanders, 2004; Jobin et al., 2019). The ethical behavior of AI systems involves diverse aspects, including safeguarding privacy, fostering fairness, promoting transparency, and embracing accountability. Given the profound impact that AI systems may exert on individuals, society, and the environment, the consideration of ethical imperatives is increasingly deemed imperative (Mittelstadt et al., 2016). Neglecting moral considerations during the development and deployment of AI systems may lead to adverse effects and social challenges (Taddeo and Floridi, 2018). Thus, for AI systems, their ethical behavior is considered a key determinant in maintaining their positive societal impact, avoiding harm and unfairness.

## 3 Implementation strategies to AI alignment

### 3.1 Mainstream methods of AI alignment for drug discovery

The key technologies utilized in the AI alignment process include reinforcement learning with human feedback (RLHF) (Knox and Stone, 2009), out-of-distribution (OOD) generalization (Ji et al., 2022; Zhuang et al., 2023; Eissa et al., 2024; Tossou et al., 2024) and OOD detection (Wang et al., 2025; Shen et al., 2024; He et al., 2024; Theunissen et al., 2025; Liu et al., 2025) techniques and visualization methods (Li et al., 2021b,a). RLHF is a subfield of reinforcement learning (RL) that incorporates human feedback into the learning process to guide and improve the performance of RL agents, whose goal is to train agents to perform specific tasks, and humans typically provide evaluation feedback or guidance to agents in the form of reward signals, criticisms, preferences, or demonstrations. This kind of human feedback helps RL agents learn more efficiently and effectively, especially in challenging domains where designing reward functions is crucial. OOD techniques (Eissa et al., 2024; Li et al., 2025; Antonluk et al., 2025) are designed to tackle the ubiquitous problem of distribution shift, wherein the distribution observed during training is different from that encountered during testing. OOD generalization refers to the ability of a model to maintain reasonable predictive ability during the testing phase for OOD data, which is new distribution samples unseen during training but are relevant to the task. It aims to learn the basic laws and transferable representations of data, rather than relying solely on the statistical characteristics of training data, in order to have stronger robustness and adaptability in open environments. OOD detection aims to automatically distinguish between input samples from the In Distribution (ID) or OOD during the model inference phase, whose core goal is to prevent the model from making high confidence erroneous predictions on unseen and distributed data, thereby improving the reliability and safety of the model in practical applications. Visualization (Li et al., 2021b, 2023, 2021a) helps to increase the interpretability of the model, which is important in understanding why the model makes such decisions and inferences. Table 1 shows the implementation strategies and mainstream methods of AI alignment for drug discovery, as well as their relationship with RICE principles. In the next section, we will introduce a technical framework and methodology aimed at studying the robustness and interpretability of neural network models, specifically addressing the first two challenges outlined in the RICE framework. This effort aims to demonstrate our conceptual approach and feasible solutions for achieving AI alignment.

**Table 1:** The implementation strategies and mainstream methods of AI alignment for drug discovery, and the implementation objectives of the RICE principles covered by each strategy and method.

**Solution**	**Direction**	**Implementation strategy**	**Methods**	**Challenges**			
				**Robustness**	**Interpretability**	**Controllability**	**Ethicality**
Graph OOD generalization and detection	Algorithm intervention	Inference environment	GraphDE (Li et al., 2022d) MoleOOD (Yang et al., 2022) GIL (Li et al., 2022c) CODI (Eissa et al., 2024) TS-DAR (Liu et al., 2025)	√		√
		Invariant learning (invariant substructure) (semantic-relevant)	iMoLD (Zhuang et al., 2023) CIGA (Chen et al., 2022) DisC (Fan et al., 2022) SCI (Li et al., 2025)	√	√		
		Disentangled representation or explainability	DIR (Wu et al., 2022) GSAT (Miao et al., 2022) GREA (Liu et al., 2022a) OOD-GNN (Li et al., 2022b)	√	√		
	Strategic intervention	Adversarial training	DAGNN (Wu et al., 2019) GNN-DRO (Sadeghi et al., 2021) GVAT (Lu et al., 2025)	√		√	
		Self-supervised learning	Pretraining-GNN (Hu et al., 2020) PATTERN (Yehudai et al., 2021) DR-GST (Liu et al., 2022b)	√			
Reinforcement learning	Policy training	Reward model (RM) Policy model (PM)	Reinforcement Learning from human Feedback (RLHF) (Touvron et al., 2023)	√		√	√
Visualization	Representation property prediction Interaction predicction	Encodings attention Mechanism adaptive graph convolution	iCAN (Weckbecker et al., 2024) Drug3D-Net (Li et al., 2021a) 3DMol-Net (Li et al., 2021b) MolLoG (Feng et al., 2024)	√	√		
		Lagrangian mechanics	LagNet (Li et al., 2023)	√	√		
	Molecular design	Geometry deep autoencoder	GEOM-CVAE (Li et al., 2024)	√	√		
Evaluation validation governance	Safety evaluations values verification AI governance	Moral values theory multi-stakeholder cooperation	Moral, legal (Erman and Furendal, 2022) Cooperative methods (Kerry et al., 2021) International governance (Tallberg et al., 2023)			√	√

The “√” stands for the main achievement goal.

### 3.2 Technical framework for exploring robustness and interpretability

**Figure 1:**
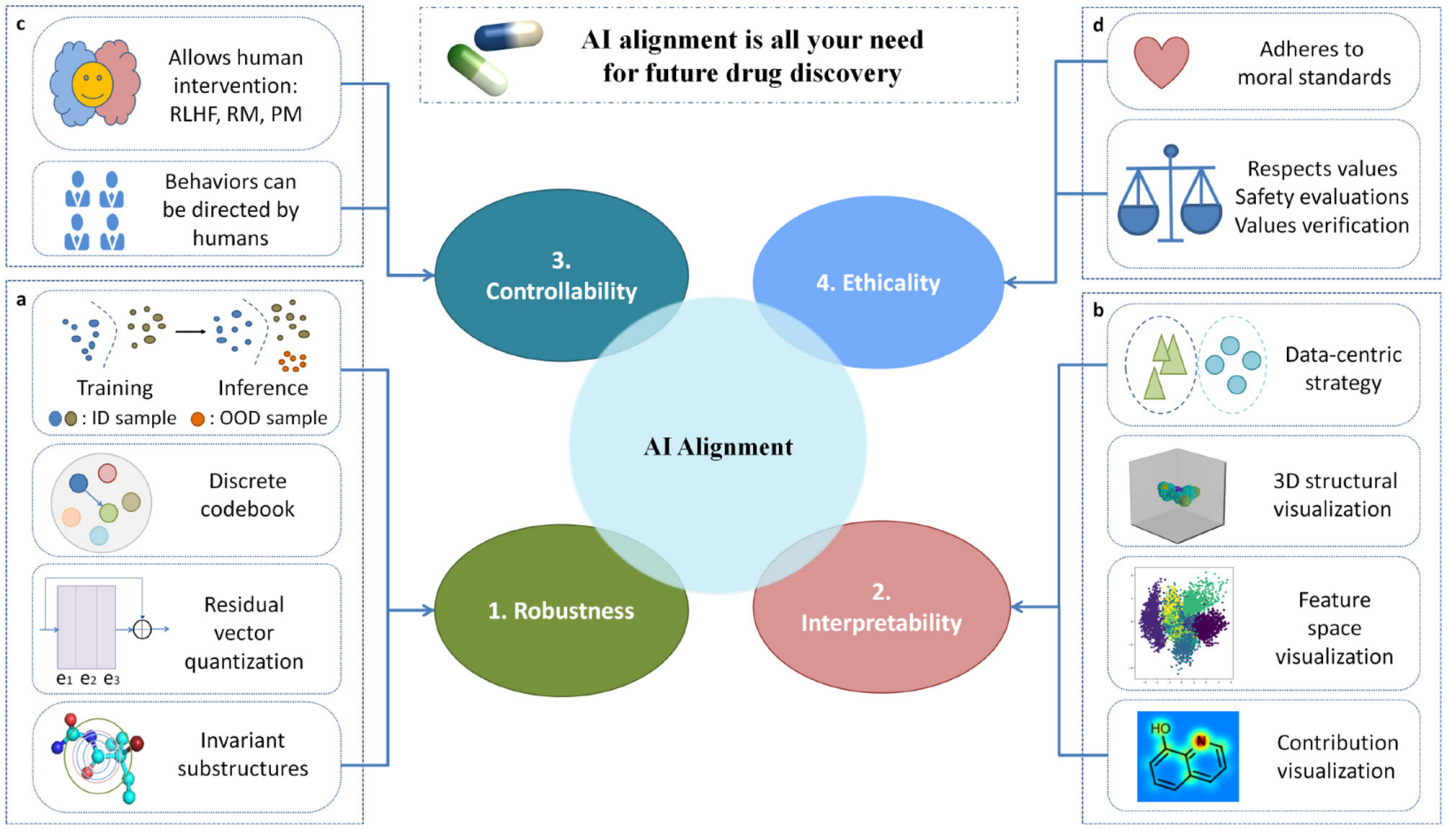
A technical framework and methodology for addressing the four challenges in AI alignment. **(a)** The out-of-distribution (OOD) module is tailored for robustness, with the capability to acquire invariance and resilience against distributional shifts. **(b)** The visualization module is specifically crafted to enhance interpretability, offering functionalities such as 3D structural visualization, feature space visualization, and contribution visualization. **(c)** The controllable module is engineered to facilitate controllability, affording opportunities for human intervention. **(d)** The module delineating social values designed for ethicality that adheres to global moral standards and upholds human values.

The fundamental concept that support AI alignment involves directing our attention toward AI technologies as a means to address the four challenges mentioned above. Figure 1 illustrates the design of our framework and methodology for addressing the four challenges in AI alignment. For each specific challenge, we have come up with the most effective approaches to response it. Specifically, in order to improve the reliability of neural network models, we suggest implementing several techniques that can enhance model robustness. Especially, we recommend focusing on AI technologies that can help models identify and detect to “out of distribution data”, meaning that models not only perform well on training data, but also work properly when encountering new samples that are different from the training data. Additionally, we suggest integrating the methods of “residual vector quantization” and “invariant substructure” into AI model. The advantage of doing so is that AI model can identify and capture key features that are consistent exist across different data, and make the model perform better and easier to understand, thereby improving its overall performance and interpretability. When addressing the challenge of interpretability in AI models, we propose a range of visualization techniques. These methods can greatly aid in understanding the inner workings of the models. Specifically, we suggest visualizing the model flowchart and molecular 3D structure, which can provide a clearer understanding of the model's architecture and the data format processed by model. Additionally, visualizing the feature space can help clarify how the model processes and distinguishes various inputs. Furthermore, by visualizing the contribution results of the target task, it is possible to gain insights into which features or components of the input data are most influential in driving the model's predictions. Then, we survey and give some insight into novel AI algorithms to address the challenges of robustness and interpretability in AI alignment for drug discovery. In recent years, graph neural networks have demonstrated impressive performance. Consequently, in this paper, we represent drug molecules using a graph structure. Within this framework, atoms are depicted as nodes, and bonds as edges. Given this representation, we aim to explore the robustness and interpretability of tasks related to molecules in non-Euclidean space.

#### 3.2.1 Suggested approaches for robustness

**Figure 2:**
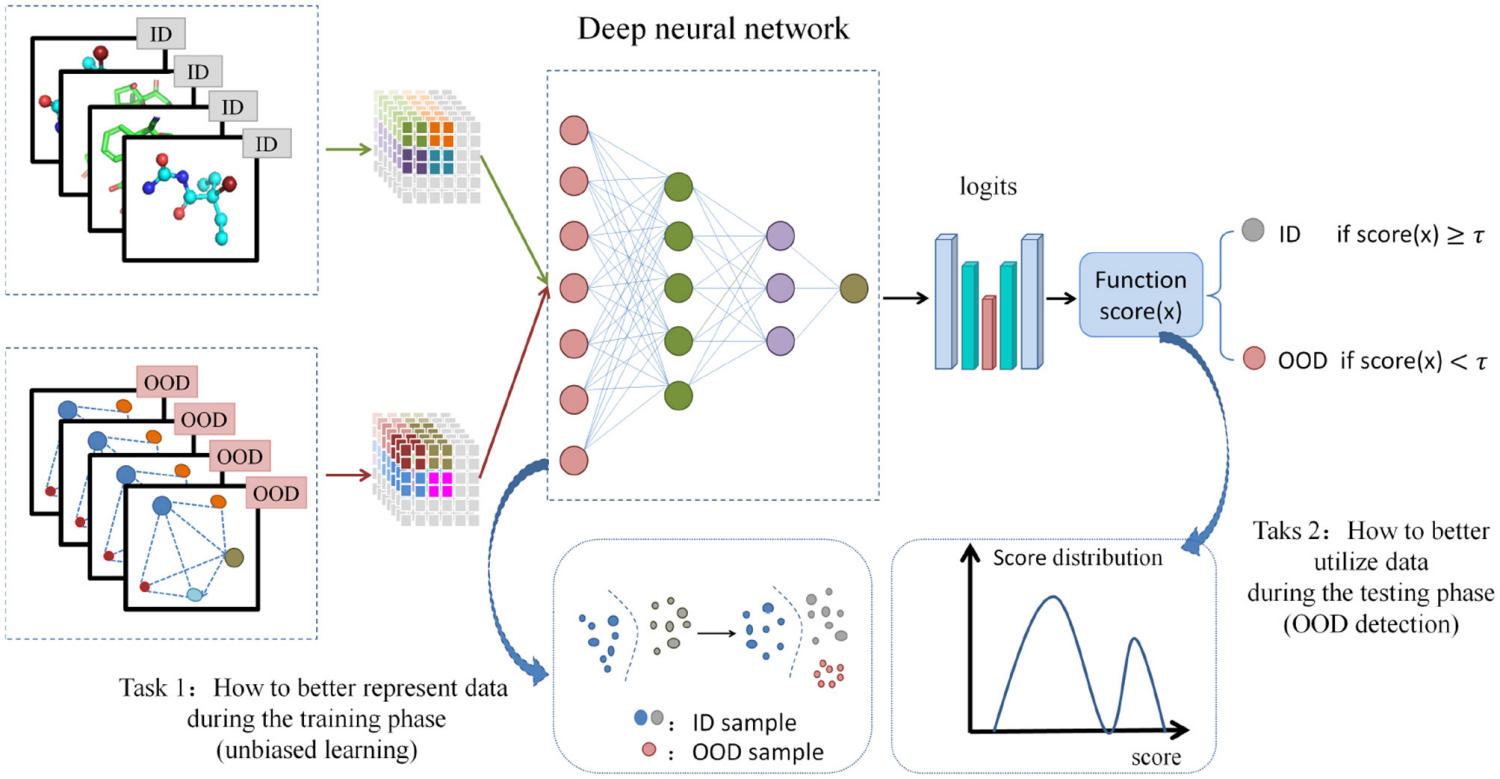
The suggested unbiased learning and out of distribution detection method. It includes two tasks: how to better represent data during the training phase (unbiased learning) and how to better utilize data during the testing phase (OOD detection).

Neural network models are always built on the assumption of independent and identically distributed (i.i.d) across training and testing data. In the field of drug discovery, when distribution shift occurs, such as molecular scaffolds (Wu et al., 2018), size (Ji et al., 2022), label noise changing on the training and testing sets, or when the i.i.d assumption is not valid, the performance of the model will be poor. As is well known, in the virtual screening process of hit recognition, the prediction model always trains on a determined target. The COVID-19 event resulted in a new target with an unprecedented data distribution, leading to a significant decrease in the performance of the prediction model when applied to this new target. OOD learning in this field focuses on handling scenarios where training and testing data present different distributions, aiming to alleviate performance degradation and improve model robustness (Zhuang et al., 2023; Muandet et al., 2013). In light of the present limitations of unbiased learning in capturing distributional shifts pertaining to both labels and feature spaces, the proposed approach endeavors to examine the interplay between unbiased learning on graphs and OOD detection within a unified latent discrete space. Subsequently, it aims to introduce a method for unbiased distributional representation of graph data and OOD detection guided by environmental variables. Figure 2 shows the overview of suggested unbiased learning and out of distribution detection method.

This method aims to utilize a binary environmental variable *e* to model both in-distribution (ID) and out-of-distribution data, where *e* = 1 denotes in-distribution data and *e* = 0 signifies biased or out-of-distribution data. Define labeled training set $D_{train}={\left\{\left(G_{train},y_{train}\right)\right\}}_{i=1}^{N_{train}}$ and test set $D_{test}={\left\{\left(G_{test}\right)\right\}}_{i=1}^{N_{test}}$, where *N*_*train*_ and *N*_*test*_ represent the number of samples in the training and test sets, respectively. Let G(V,E) be a graph with adjacency matrix $A=\left\{a_{uv}|u,v\in V\right\}$ and initial node features *X* = {*x*_*v*_} for v∈V, where V is nodes set and E is edges set. Our objective is to develop a predictive model *f*_η_:*G* → ŷ capable of accurately forecasting labels within $D_{test}$. Let $L\left(f_{\eta }^{e}\left(G\right),y\right)$ as the loss function on sample (*G, y*) under environment *e*, then the empirical risk minimization (ERM) (Cherkassky, 1997) can be defined as


(1) $$\begin{array}{l} \underset{\eta }{\min}{𝔼}_{e\sim p\left(e\right)}[{𝔼}_{\left(G,y\right)\sim P\left(G,y|\text{e}=e\right)}\left[L\left(f_{\eta }^{e}\left(G\right),y\right)\right]] \end{array}$$

where $L\left(f_{\eta }^{e}\left(G\right),y\right)$ is a constant when *e* = 0 holds, *p* denotes the probability distribution and satisfies the following condition: *p*(*G, y*|*e*) = *p*(*G*|*e*)*p*(*y*|*G, e*). Furthermore, to identify OOD samples within the test set, it is imperative to design an OOD detector *dect*, including predictive model *f*_η_, score function *score*, threshold χ. The detector *dect* can be defined as:


(2) $$\begin{array}{l} dect\left(G;f_{\eta },score,\chi \right)=\{\begin{array}{c} 0\;\left(OOD\right),ifscore\left(G;f_{\eta }\right)\leq \chi \\ 1\;\left(ID\right),ifscore\left(G;f_{\eta }\right)>\chi \end{array} \end{array}$$

Through this approach, the model engages in unbiased learning during the training phase and additionally performs out-of-distribution detection during the testing phase of downstream tasks. This strategy effectively mitigates the negative impact of out-of-distribution samples on the model's performance, consequently enhancing its efficacy and robustness. Hence, the proposed method exemplifies our strategic approach and investigational efforts toward bolstering the robustness of the model, constituting a modest stride in the direction of fortifying its resilience. In the field of drug discovery, in terms of OOD generalization, CODI (Eissa et al., 2024) is a contextual OOD integration method designed to generates synthetic data by incorporating unrepresented sources of variation observed in real-world applications into a given molecular fingerprint dataset. By augmenting the dataset with OOD variance, CODI enhances the ability of machine learning models to generalize to samples beyond the original training data, thereby reducing the reliance on extensive experimental data collection. Tossou et al. present a rigorous method (Tossou et al., 2024) to investigate molecular OOD generalization in the field of drug discovery. This method uses covariate changes to quantitatively quantify the distribution changes of sample distance to the training set encountered during actual deployment, which can lead to performance degradation of up to 60% and uncertainty calibration degradation of up to 40%. In terms of OOD detection, PGR-MOOD (Shen et al., 2024) is a molecular OOD detection method by using an auxiliary diffusion model, which compares similarities between input molecules and reconstructed graphs. Due to the generative bias toward reconstructing in-distribution training samples, the similarity scores of OOD molecules will be much lower to facilitate detection. GDDA (He et al., 2024) is a novel two-phase method of graph disentangled diffusion augmentation, aimed to disentangle graph representations into semantic factors and style factors by using a distribution-shift-controlled score-based diffusion model. Theunissen et al. evaluate six OOD detection methods to demonstrate OOD detection performance in both synthetical and real-world application settings (Theunissen et al., 2025), specifically in the context of single-cell transcriptomics annotation. Liu et al. proposed TS-DAR (Liu et al., 2025), a transition state identification method based on dispersion and variational principle regularized neural networks. TS-DAR is a deep learning framework inspired by OOD detection in trustworthy artificial intelligence, aimed at understanding protein conformational changes. Unlike traditional Molecular Dynamics (MD) simulations, TS-DAR leverages deep learning techniques to identify transition states, offering a novel approach to studying dynamic molecular processes.

#### 3.2.2 Suggested approaches for interpretability

It is widely acknowledged that neural networks are often referred to as black box models primarily due to the inherent challenge in intuitively understanding and elucidating their internal operating mechanisms. Neural networks typically comprise a multitude of neurons and layers, learning patterns and features of input data through intricate weight adjustments during training. Given the intricate internal architecture of neural networks, elucidating the precise relationship between individual neurons and weights proves challenging, thereby impeding the intuitive explanation of neural network decision-making processes. Despite neural networks' capability to undergo training on extensive datasets and yield highly accurate predictions, their decision-making processes often remain opaque and defy straightforward explanation. In the realm of drug discovery, the interpretability of models assumes paramount importance, as it fosters patient trust in medical diagnoses and facilitates the provision of comprehensive disease treatment by healthcare professionals.

**Figure 3:**
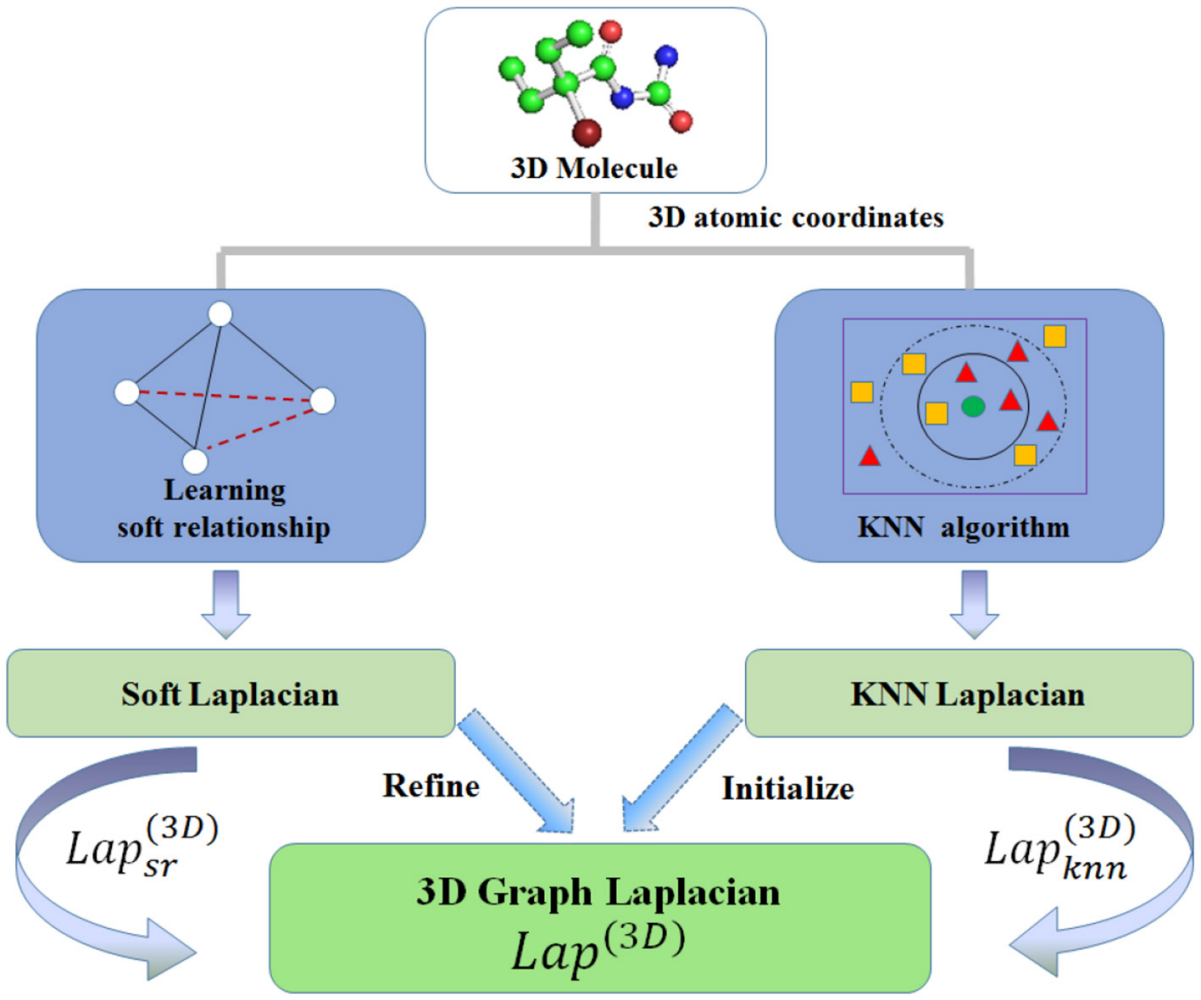
3DMol-Net: a 3D garph Laplacian, which is composed of KNN Laplacian and soft Laplacian.

Drug molecules are always represented as graph structures. Rotation invariance-based 3DMol-Net (Li et al., 2021b) model demonstrates the interpretability by learning three-dimensional (3D) soft relation and K-nearest neighbors (KNNs) relation in 3D space, subsequently constructing 3D graph Laplacian, then building rotation-invariant map (RIM) with attention mechanism. This process can be expressed as follows:


(3) $$\begin{array}{l} Lap_{sr}^{\left(3D\right)}=I_{n}-{D_{sr}}^{-\frac{1}{2}}F_{sr}{D_{sr}}^{-\frac{1}{2}} \end{array}$$

(4) $$\begin{array}{l} Lap_{knn}^{\left(3D\right)}=I_{n}-{D_{knn}}^{-\frac{1}{2}}F_{knn}{D_{knn}}^{-\frac{1}{2}} \end{array}$$

(5) $$\begin{array}{l} Lap^{\left(3D\right)}=Lap_{knn}^{\left(3D\right)}+\;\alpha Lap_{sr}^{\left(3D\right)} \end{array}$$

where $F_{sr}$ and *D*_*sr*_ denote the soft relationship matrix and its degree matrix, respectively. Similarly, $F_{knn}$ and *D*_*knn*_ denote the KNN relationship matrix in 3D space and its degree matrix, respectively. $Lap_{knn}^{\left(3D\right)}$ and $Lap_{sr}^{\left(3D\right)}$ and represent Laplacian matrices under KNN relationships and soft relationships, respectively. Finally, the proposed 3D graph Laplacian matrix *Lap*^(3*D*)^ can be obtained using Equation 5 with a hyperparameter α. As shown in Figure 3, *Lap*^(3*D*)^ is initialized by KNN Laplacian $Lap_{knn}^{\left(3D\right)}$ and refined by soft Laplacian $Lap_{sr}^{\left(3D\right)}$. Then define *RIM* as a function of rotation-invariant map, *V*_*coord*_ as 3D coordinates vector for each atom. The final rotation invariance-based geometric representation *F*_*ri*_*geom*_ for molecules can be calculated as:


(6) $$\begin{array}{l} F_{ri\text{\_}geom}=RIM\left(Lap^{\left(3D\right)},\;V_{coord}\right) \end{array}$$

Furthermore, attention *att*_*mn*_ can be formulated as:


(7) $$\begin{array}{l} att_{mn}=\frac{exp\left(LeakyRelu\left(W\cdot \left[h_{m},h_{n}\right]\right)\right)}{\sum_{n\in N\left(m\right)}exp\left(LeakyRelu\left(W\cdot \left[h_{m},h_{n}\right]\right)\right)} \end{array}$$

where *m* is the target atom, *n* is the neighbor node, and *h*_*m*_, *h*_*n*_ represent the state vectors of atom *m* and *n*, respectively. *W* denotes trainbale weight matrix. *LeakeRelu* denotes nonlinear activation function.

**Figure 4:**
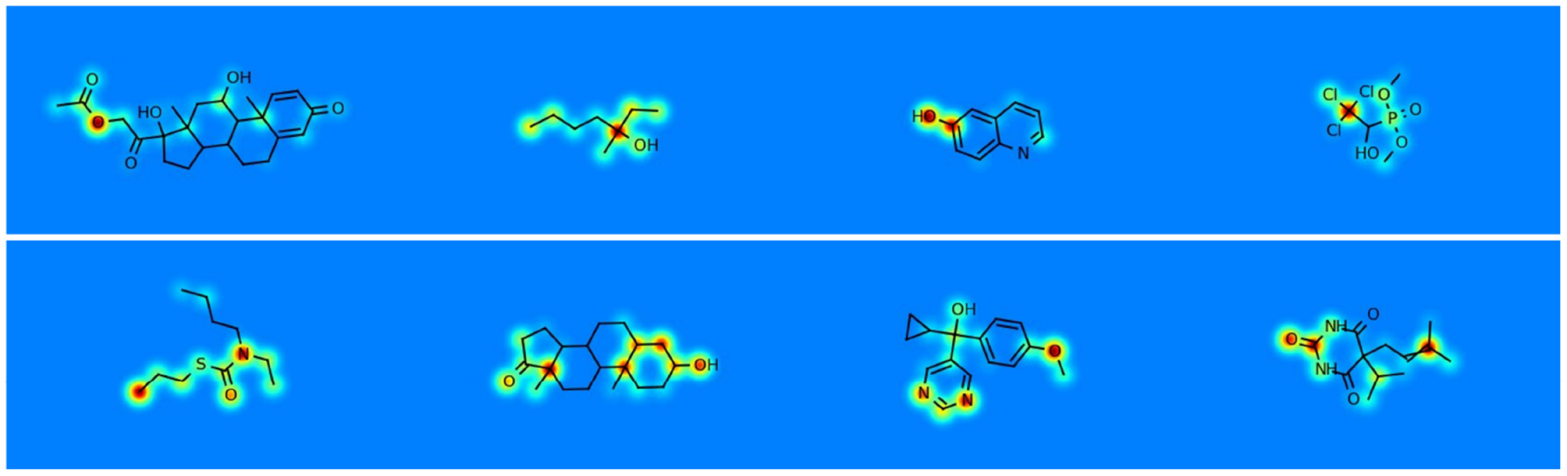
Visualizing the contribution of atoms to molecule-level predictive tasks, that is shown using jet colormap with range [0.25, 1.0]. Red signifies the most significant contribution, blue denotes the least contribution, while yellow and green lie in the intermediate range.

**Figure 5:**
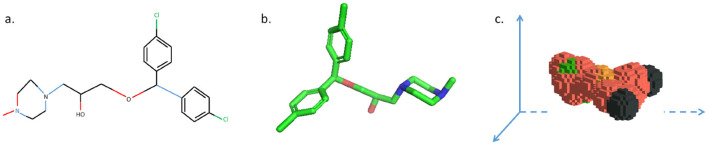
Three represents for the same drug molecule. **(a)** 2D molecular structure. **(b)** 3D molecular structure. **(c)** 3D voxel structure automatically modeled in 3D space using python language.

The 3DMol-Net leverages adaptive graph convolutional networks to proficiently acquire the 3D molecular representations, showcasing commendable efficacy in predicting molecular properties. Moreover, the proposed model exhibits notable interpretability in discerning and explicating the predicted outcomes. As shown in Figure 4, hydrophilic groups exhibit a more pronounced influence on predicting water solubility in the ESOL dataset, with their contribution to molecular features playing a decisive role in the final prediction. Furthermore, the group most pertinent to predicting the activity of inhibiting HIV replication demonstrates a heightened contribution to graph-level representation. Additionally, 3D structural visualization greatly improves the interpretability of AI models within the context of drug discovery. The 3D structural attributes of small molecules and proteins play a crucial role in determining their biochemical functions and activity predictions. These 3D characteristics predominantly dictate both the properties of drugs and the binding characteristics of their respective targets. Therefore, Li et al. (2021a) conducted visualization of 3D voxel representations of molecules within 3D space, with distinct colors denoting various atoms, as shown in Figure 5. This visualization technique affords a more intuitive comprehension of the three-dimensional microstructure of molecules, thereby exerting a discernible influence on the prediction of drug-related interactions, such as drug-drug interaction (DDI) and compound protein interaction (CPI). In addition, iCAN (Weckbecker et al., 2024) aims to covercome the constraints of machine learning models that typically rely on structured and rigid input formats. It encodes the neighborhoods of carbon atoms using a counting array, enhancing the effectiveness of the generated representations for machine learning tasks. By producing interpretable molecular encodings, iCAN method facilitates the comparison of molecular neighborhoods, the detection of recurring patterns, and the visualization of important features through heat maps. MolLoG (Feng et al., 2024) is a molecular deep interpretability method that establishes a bridge between local features and global representations, aiming to enhance the prediction of drug-target interactions. MolLoG comprises local feature encoders (LFE) and global interactive learning (GIL) modules, offering biologically relevant interpretations for the predictions generated by black-box models.

**Figure 6:**
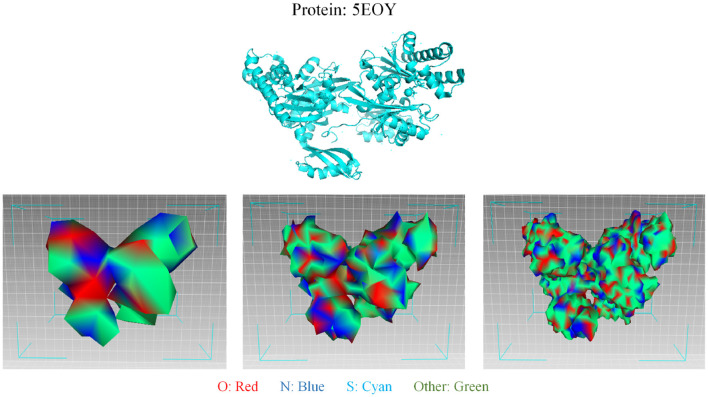
Visualization of the 3D mesh structure of protein surfaces. The first line depicts the original folding of the protein 5EOY, visualized using PyMOL. The second line illustrates the modeling of the 5EOY surface utilizing three-dimensional (3D) mesh, which encompasses abundant geometric information crucial for deriving an effective protein characterization. From left to right in the second line, the mesh resolution progressively increases, accompanied by an increasing number of vertices in the 3D mesh. The different colors in the mesh represent different surface features. In AIGC-related tasks, such as drug design and the prediction of compound-protein binding pockets, this visualization method and underlying representation techniques demonstrate commendable robustness and interpretability.

As for protein 3D representation, since the 3D conformation of a protein dictates its function, the 3D structure of proteins is foundational to understanding their role in biological systems. The specific arrangement of amino acids determines how a protein interacts with other molecules, including substrates and inhibitors. Consequently, insights into protein structure can reveal mechanisms of action and inform the design of new drugs. Furthermore, the stability and dynamics of protein structures are integral to their functionality. Changes in a protein's conformation can significantly affect its activity and interactions. Thus, elucidating these structural nuances is essential for predicting how proteins behave under various conditions. Following GEOM-CVAE (Li et al., 2024), the 3D mesh structure of protein surfaces can be visualized in Figure 6, which encompasses abundant geometric information crucial for deriving an effective protein characterization. The different colors in the mesh represent different surface features. The process of mesh simplification is illustrated from right to left. The simplification of protein 3D mesh can also be regarded as the graph sampling and graph pooling in graph neural network. In tasks associated with AI-Generated Content (AIGC), such as drug design and the prediction of compound-protein binding pockets, this visualization method and underlying representation techniques demonstrate commendable robustness and interpretability.

### 3.3 Strategies for incorporating human values

AI systems have penetrated into various aspects of our lives and careers, that are bringing us numerous conveniences. It is worth noting that advanced AI models, such as AIGC large language systems, possess the ability to independently decompose complex tasks into manageable subtasks and execute decisions without human intervention. The emergence of AIGC has brought substantial advantages, especially in improving productivity, tailoring services according to personal preferences, cultivating creativity and breakthroughs, and promoting industrial progress. However, on the other hand, these are countless inherent potential risks associated with AI systems. Empirical research emphasizes the potential capacity of AI systems to pose a threat to global security (Turchin and Denkenberger, 2020; Ji et al., 2024). For instance, the initial iterations of the GPT-4 model, as identified by OpenAI, exhibited a series of dangerous behaviors, including spreading misinformation, manipulating public emotions, and even formulating novel biochemical substances (OpenAI, 2023a). Furthermore, investigations by Urbina et al. (2022) emphasize the potential health risks posed by AI systems in domains such as drug discovery and synthetic biology. The AIGC models tailored for drug design have generated an astonishing 40,000 toxic molecules, whose synthesis and introduction into the human body could potentially trigger significant disasters.

In addition, artificial intelligence has also raised issues such as employment and economic inequality, privacy breaches and security risks, algorithmic bias and discrimination, and ethical concerns (Taddeo and Floridi, 2018; Jobin et al., 2019; Floridi and Cowls, 2019). In the absence of adequate regulation and governance, artificial intelligence systems have the potential to pose catastrophic risks to humanity, that may even endanger human survival (Yu et al., 2018). We must maintain a profound sense of responsibility toward AI alignment. Here are several strategies for integrating human values:

- Technology Integration: The task of AI developers and researchers is to consider AI alignment issues right from the beginning of AI system design. This requires careful design of artificial intelligence algorithms, models, and training protocols, with a focus on prioritizing security, transparency, and consistency with human values. Here are some examples of how human values can be applied in AI design, which will help to bridge theory with practice. For instance, fairness can be applied to AI-driven drug discovery by incorporating fairness metrics to mitigate bias in molecular property prediction tasks, thereby reducing potential biases generated by imbalanced training data (Chen et al., 2024; Salmi et al., 2024). Transparency may be achieved by employing interpretable graph neural networks or attention mechanisms in candidate drug design, which allow researchers and clinicians to understand how candidate molecules are prioritized (Li et al., 2021b; Fang et al., 2023). Accountability can be strengthened through the integration of bias detection and monitoring protocols during model training and validation, which ensures that systematic errors are identified early and addressed (Antonluk et al., 2025; Liu et al., 2024). Together, these practices demonstrate how to embed human values into the workflow of artificial intelligence, that enables drug discovery systems to be not only technically effective but also ethically aligned and socially responsible.
- Policy Regulation: Governments play a crucial role in formulating and managing the regulations for the development and deployment of artificial intelligence. By developong responsible AI policies, guidelines, ethical frameworks, interdisciplinary collaboration and ethical oversight and accountability, we ensure that artificial intelligence systems uphold human values and adhere to ethical standards. For example, the European Union's AI Act proposes a comprehensive legal framework that classifies AI applications based on risk and mandates requirements such as transparency, human oversight, and accountability for high-risk systems, which include applications in healthcare (European Commission, 2021). Similarly, the U.S. Food and Drug Administration (FDA) has released guidelines on AI/ML-based Software as a Medical Device (SaMD), which emphasize continuous learning, performance monitoring, and transparency to ensure patient safety and ethical compliance (U.S. Food and Drug Administration, 2021). These measures demonstrate how regulatory measures can effectively safeguard the fairness, reliability, and societal trust in the application of AI for drug discovery.
- Public Engagement: Collaboration on a global scale is crucial for raising public awareness about the challenges and impacts of AI alignment. It can facilitate mutual supervision and ensures that different perspectives are taken into account throughout the alignment process by encouraging public participation in discussions on artificial intelligence ethics. Global cooperation and public consultation are crucial for ensuring AI alignment in reflecting different perspectives and social values. The OECD Principles on Artificial Intelligence and other international initiatives provide examples of how multi stakeholder governance can promote responsible AI practices (OECD Legal Instruments, 2019). Similarly, the European Union's Artificial Intelligence Act explains how transparent participation mechanisms can incorporate public opinion into policy frameworks (European Commission, 2021). The participatory design approaches further emphasize that involving end-users and affected communities in the early stages of the process can enhance inclusivity and legitimacy (Vines et al., 2013).

## 4 Global developments in AI alignment for drug discovery

Countries have made certain progress and achievements in aligning AI with the field of drug discovery. The United States has always been at the forefront of drug discovery, with its AI aligned strategies mainly focused on funding research, policy-making, and regulation. For example, the National Institutes of Health (NIH) and the Food and Drug Administration (FDA) have funded many AI projects for drug discovery and developed relevant policy guidance (Uddin et al., 2025; National Institutes of Health (NIH), 2023). In addition, some large pharmaceutical companies in the United States, including Pfizer and Merck, are actively exploring the application of AI in drug discovery (XtalPi, 2025; Merck, 2023). The Chinese government regards healthcare as one of the important development areas and promotes the development of AI in the field of drug discovery through funding research projects, formulating policies, and strengthening international cooperation. Recently, China has proposed the concept of “new quality productive forces”, which are advanced productive forces led by innovation and in line with the new development concept, ultimately achieving harmonious coexistence between humans and nature (The Central People's Government of the People's Republic of China, 2024). The European Commission has funded many AI projects for drug discovery through institutions such as the European Innovation Commission, and has proposed strategic goals to promote pharmaceutical innovation and drug discovery (European Innovation Council, 2022). Other countries, such as Canada, the United Kingdom, Germany, etc., have also conducted some research and practice on AI alignment in the field of drug discovery. Some research institutions and pharmaceutical companies are also actively exploring the application of AI in drug discovery (UK Biobank, 2025). In contrast, the United States emphasizes regulatory leadership and industry adoption, while China emphasizes state driven innovation and integration into broader socio-economic strategies, and the European Union prioritizes cross-border cooperation and ethical governance frameworks. These different methods have demonstrated their respective advantages (Blanco-González et al., 2023). Future research and policies should integrate the effectiveness of these different strategies and promote global cooperation to address ethical and practical challenges, which will enable responsible and influential artificial intelligence to play a role in drug discovery.

## 5 Conclusion

Drawing on insights from the fields of computer science, and pharmacology, the paper explores the potential benefits of human-centered AI alignment in drug discovery, such as enhanced safety, effectiveness, and accessibility of pharmaceutical interventions. By prioritizing human values and societal well-being, AI-driven drug discovery programs can better meet the needs and preferences of patients, clinicians, and other stakeholders. Subsequently, the paper focuses on the challenges faced by artificial intelligence alignment and measures to address these challenges, incorporating human values into the design and implementation of drug discovery. AI alignment not only emphasizes the integration of ethical principles, stakeholder engagement, and interdisciplinary collaboration throughout the entire AI development lifecycle, but also recommends the use of robustness, transparent and interpretable AI models which incorporate different perspectives in algorithmic decision-making, and establish sustained ethical oversight and accountability mechanisms.

Looking ahead, we should conduct further research on how to achieve deep coupling between artificial intelligence and human values throughout the entire drug discovery process. On the one hand, it is necessary to explore new interdisciplinary collaboration models that integrate ethics, clinical medicine, pharmacology, and artificial intelligence algorithm research more closely to promote the integration and innovation of interdisciplinary knowledge. On the other hand, we should strengthen research on the interpretability and causal inference ability of artificial intelligence models, which not only provide efficient prediction results, but also provide traceable scientific basis for drug mechanism research and clinical decision-making. In addition, we should also focus on studying issues of fairness and universality, ensuring that artificial intelligence systems can maintain stable performance and fair results when facing different populations, disease types, and medical environments. At the practical level, it is recommended to establish a long-term ethical supervision and evaluation framework, which combined with dynamic regulatory mechanisms and open science principles, to ensure the safe, controllable, and transparent development of artificial intelligence technology. Through continuous exploration in the above directions, it is expected to promote the landing and popularization of human-centered artificial intelligence in drug discovery, thereby promoting the sustainable development of the healthcare industry.

In conclusion, we advocates for a paradigm shift toward human-centered AI alignment in drug discovery. Researchers, policy makers, and industry stakeholders should prioritize ethical considerations and societal impact when developing and applying AI systems. Only in this way can the application of artificial intelligence in biomedical research truly serve human well-being. By aligning AI with human values, it can not only play a huge role in promoting the development of healthcare, but also ensure that the benefits brought by technology can be fairly distributed. This can also make the entire process more ethical. All in all, AI alignment is all your need for future drug discovery.
